# Sex-specific determinants of serum 25-hydroxyvitamin D_3_ concentrations in an elderly German cohort: a cross-sectional study

**DOI:** 10.1186/1743-7075-12-2

**Published:** 2015-01-14

**Authors:** Alexandra Jungert, Monika Neuhäuser-Berthold

**Affiliations:** Institute of Nutritional Science, Justus-Liebig-University, Goethestrasse 55, D-35390 Giessen, Germany

**Keywords:** 25-Hydroxyvitamin D, Determinants, Lifestyle, Body composition, Diet, Socioeconomic factors, Elderly

## Abstract

**Background:**

Considering the suggested link between vitamin D insufficiency and several chronic diseases, attention should be given to approaches for improving vitamin D status. Elderly subjects are regarded as a high-risk group for developing an insufficient vitamin D status. Socioeconomic, dietary, lifestyle and environmental factors are considered as influencing factors, whereupon sex differences in predictors of vitamin D status are rarely investigated. The purpose of this study is to identify the main predictors of serum 25-hydroxyvitamin D_3_ [25(OH)D_3_] concentrations in elderly subjects by taking into account potential sex differences.

**Methods:**

This is a cross-sectional study in 162 independently living German elderly aged 66 to 96 years. Serum 25(OH)D_3_ concentrations were assessed by an electrochemiluminescence immunoassay. Multiple regression analyses were performed to identify predictors of 25(OH)D_3_ concentrations stratified by sex.

**Results:**

Median 25(OH)D_3_ concentration was 64 nmol/L and none of the subjects had 25(OH)D_3_ concentrations < 25 nmol/L. In women, intact parathyroid hormone (iPTH) (*β* = -0.323), % total body fat (*β* = -0.208), time spent outdoors (*β* = 0.328), month of blood sampling (*β* = 0.229) and intake of vitamin D supplements (*β* = 0.172) were the predominant predictors of 25(OH)D_3_, whereas in men, iPTH (*β* = -0.254), smoking (*β* = -0.282), physical activity (*β* = 0.336) and monthly household net income (*β* = 0.302) predicted 25(OH)D_3_ concentrations. The final regression models accounted for 30% and 32% of the variance in 25(OH)D_3_ concentrations in women and men, respectively.

**Conclusion:**

The findings indicate that 25(OH)D_3_ concentrations are influenced by body composition, month of blood sampling, economic factors, lifestyle, supplement intake and iPTH, but may not be associated with age, sex, dietary factors, kidney function and presence of selected chronic diseases in community-dwelling elderly. Furthermore, our results provide evidence for sex-specific determinants of the vitamin D status, which ought to be considered for preventive strategies.

## Background

Vitamin D insufficiency is a widely recognised public health problem, especially in elderly subjects [1]. The decrease in the circulating concentration of 25-hydroxyvitamin D [25(OH)D] – the established biomarker of vitamin D status – with advancing age seems to be accompanied by a gradual rise in chronic diseases. Experimental and epidemiological evidence suggests that vitamin D deficiency is involved in the pathogenesis of many age-related chronic diseases, including cancer [2], cardiovascular disease [3] and diabetes mellitus [4]. In consideration of the assumed pleiotropic functions of vitamin D beyond its well-known role in calcium homeostasis, public health strategies to improve vitamin D status in the elderly population are warranted. However, uniform thresholds for classifying serum 25(OH)D concentrations into adequacy and insufficiency are not yet established. The most common used cut-off values to diagnose vitamin D insufficiency are 25(OH)D concentrations below 50 nmol/L [5] and below 75 nmol/L [6].

To establish efficient prevention measures concerning vitamin D insufficiency and to distinguish subjects who would benefit most from a vitamin D screening or supplementation, factors that influence vitamin D status in the elderly have to be identified. Although low sun exposure and a decline in subcutaneous vitamin D synthesis capacity are considered as main contributing factors regarding low 25(OH)D concentrations in the elderly [1], these parameters explained only a small part of the variance in vitamin D status in previous studies [7]. This may be due to the difficulty in obtaining accurate measures of UVB exposure [8]. Apart from a low sun exposure, an insufficient vitamin D status has been linked to advanced age [9, 10], female sex [9–11], low vitamin D intake [11, 12], obesity [9, 11–14], sedentary lifestyle [11, 12, 14], smoking [9, 11, 13, 14], use of specific medicines [15–17] and the presence of chronic diseases [9, 15]. However, most previous studies examined only a small number of potential influencing factors simultaneously. Economic aspects, dietary factors, health status and the use of medicines were rarely considered. Furthermore, although there is some evidence that the predictors of serum 25(OH)D concentrations differ by sex [7, 11, 13, 18], sex-specific analyses are especially scarce for the elderly population. Therefore, the purpose of the present study is to identify determinants of the serum 25-hydroxyvitamin D_3_ [25(OH)D_3_] concentrations in independently living elderly subjects by taking into account socioeconomic, dietary, lifestyle and health aspects in addition to factors, such as age, month of blood sampling, body composition, kidney function and concentrations of intact parathyroid hormone (iPTH).

## Methods

### Ethics statement

This study was conducted according to the guidelines laid down in the Declaration of Helsinki and the research protocol was approved by the Ethical Committee of the Faculty of Medicine at the Justus-Liebig-University of Giessen, Germany. All participants provided written informed consent before examinations.

### Study design and subjects

The present investigation based on the follow-up in 2008 of the longitudinal study on nutrition and health status of senior citizens in Giessen (GISELA study), Germany located at northern latitude of 50.6°, in which 275 subjects participated. Investigations of the GISELA study took place at annual intervals since 1994 and at biannual intervals since 1998. Study participants were volunteers and recruited by physicians, notices, senior citizens’ meetings, advertisements in local newspapers and through subjects who were already participants. Inclusion criteria for enrolment in the study were an age of at least 60 years and physical mobility. Investigations have taken place in the Institute of Nutritional Science in Giessen from July to October.

Subjects with incomplete data on biochemical parameters, body composition, lifestyle factors, including time spent outdoors, physical activity and smoking, dietary assessment, household net income and chronic kidney disease were excluded (*n* = 105) as well as subjects who suffered from chronic kidney disease (*n* = 5). Three further subjects were excluded to minimise potential influence by outlying 25(OH)D_3_ values and to obtain normal distribution of residuals. The final study sample consisted of 113 women and 49 men.

### Biochemical analyses

Fasting blood samples were collected in 2008 from July to September, and serum aliquots were stored at -70°C until further analyses. Serum 25(OH)D_3_ and iPTH were measured by an electrochemiluminescence immunoassay (Roche Diagnostics GmbH, Mannheim, Germany) in the Limbach Laboratory, Heidelberg, Germany. The lower limits of quantification were 10.0 nmol/L for 25(OH)D_3_ and 0.127 pmol/L for iPTH, respectively [19, 20]. The coefficients of variation for the total analytic precision of the assays were ≤ 9.9% for 25(OH)D_3_ and ≤ 5.9% for iPTH, respectively [19, 20]. The laboratory performing the 25(OH)D_3_ analyses took part in the Vitamin D External Quality Assessment Scheme. The 25(OH)D_3_ assay has been standardised against liquid chromatography-tandem mass spectrometry (LC-MS/MS) [19].

Serum 25(OH)D_3_ concentrations below 25.0 nmol/L and between 25.0 and 49.9 nmol/L were considered as deficient and insufficient vitamin D status, respectively. Due to the ongoing discussion about optimal 25(OH)D_3_ values, two cut-off values were used to diagnose vitamin D sufficiency: ≥ 50 nmol/L according to the Institute of Medicine [5] and ≥ 75 nmol/L according to the Endocrine Society [6].

Serum creatinine was determined by photometric detection using the Jaffé reaction (Shimadzu UV-160A) [21]. The estimated glomerular filtration rate (eGFR) was calculated as an indicator of kidney function using the simplified Modification of Diet in Renal Disease study (MDRD) formula: eGFR (mL/min/1.73 m^2^) = 186 × (serum creatinine mg/dL)^–1.154^ × (age)^–0.203^ × 0.742 (if female) [22].

### Anthropometric data and body composition

Weight and height were assessed in a standardised way with subjects in light clothing without shoes, using a calibrated scale with an integrated stadiometer, and body mass index (BMI) (kg/m^2^) was calculated. Percentage total body fat (% TBF) was determined by a single-frequency (50 kHz) bioelectrical impedance analyser (Akern-RJL BIA 101/S®; Data Input, Frankfurt, Germany) and the formula of Roubenoff et al. [23].

### Data on socioeconomic and lifestyle factors

Participants were asked to complete a self-administered questionnaire on health, socioeconomic and lifestyle characteristics, such as monthly household net income, smoking status, physical activity, time spent outdoors, intake of medicines and disease history (including for instance hypertension, cardiac disease, dyslipidaemia, diabetes mellitus, osteoporosis, cancer, liver/gall bladder disease and disease of the urinary tract/kidney).

Study participants were asked to record their current time spent on different physical activities in hours per week. The energy expenditure for the respective physical activities was calculated by multipliers for resting metabolic rate (RMR) according to the World Health Organization (WHO) [24]. RMR was measured by an open-circuit indirect calorimeter (Deltatrac™ MBM-100, Hoyer, Bremen, Germany) using the equation of Weir [25]. Total energy expenditure was calculated by the sum of the energy expenditure for the respective physical activities. The physical activity level (PAL) of each participant was computed by dividing the total energy expenditure by the RMR [26].

Sunlight exposure was indirectly assessed by the following question: "Please estimate, how much time do you currently spend outdoors in minutes per day?". In the follow-up in 2008, blood samples of the GISELA subjects were obtained from July to September. Mean monthly UV indices assessed by a weather station located in the area of Giessen (city: Langen, latitude of 50°N) ranged in 2008 from six in June (data for July were not available) to five in August to three in September [27]. Because in 2008 the follow-up started on July the 29th, the examination months July (*n* = 18) and August (*n* = 84) were combined for the present analysis.

### Dietary assessment

Dietary intake of vitamin D, calcium, phosphorus and alcohol was determined using a three-day estimated dietary record consisting of 146 food items and beverages, which was developed and validated for the GISELA study [28]. Data on vitamin D and calcium supplement intake were collected via a self-administered questionnaire.

### Statistical analyses

Continuous data are expressed as median and 5th and 95th percentiles due to the presence of non-normally distributed variables. Descriptive characteristics were compared between groups via Mann-Whitney *U* test for continuous variables. Chi-square test or Fisher’s exact test was used to assess differences in proportions.

At first, we analysed whether self-reported lifetime histories of selected diseases and/or current medication influenced 25(OH)D_3_ concentrations by using Mann-Whitney *U* test and simple and stepwise multiple linear regression analyses adjusting for sex, age, iPTH, % TBF, month of blood sampling, nutrient intake and lifestyle factors, respectively. Diseases or medication which showed associations with 25(OH)D_3_ concentrations were considered in subsequent multiple regression analyses.

Multiple regression analysis with backward elimination was used to investigate the relationships between serum 25(OH)D_3_ as continuous outcome variable and age, sex, body composition, eGFR, iPTH, month of blood sampling, nutrient intake, lifestyle factors, household net income and, if appropriate, disease histories and/or current medication as possible independent variables. Variables with a *P*-value ≤ 0.100 were allowed to remain in the model. Variance inflation factors were ≤ 3 and tolerance statistics were > 0.3, indicating that there was no multicollinearity between parameters. To evaluate whether sex was an effect modifier as regards the assumed associations of lifestyle variables, iPTH, eGFR, body composition and income with 25(OH)D_3_, interaction terms (sex × time spent outdoors, sex × PAL, sex × smoking, sex × iPTH, sex × eGFR, sex × % TBF and sex × household net income) were investigated. In the multiple regression analyses stratified by sex, only factors which have been proved as determinants of the 25(OH)D_3_ concentration in the combined study population or factors for which an interaction by sex was observed were considered as independent variables. Finally, we performed a sensitivity analysis by excluding subjects with an eGFR ≤ 30 mL/min/1.73 m^2^ (*n* = 3). Statistical analyses were conducted with SPSS® 22.0 for Windows (IBM®, Chicago, USA). Significance level was set at *P* < 0.05, and all tests were two-tailed.

## Results

### Characteristics of the study subjects

Descriptive characteristics of the subjects are presented in Table 1. Median 25(OH)D_3_ concentration was 64 nmol/L (range: 30 – 98 nmol/L). None of the subjects had a severe vitamin D deficiency (25(OH)D_3_ < 25 nmol/L). Insufficient 25(OH)D_3_ concentrations (< 50 nmol/L) were found in 21.2% of the females and 14.3% of the males (19.1% combined) and concentrations ≥ 75 nmol/L were evident in 19.5% of the females and 18.4% of the males (19.1% combined). With regard to these results, no sex differences were found.

**Table 1:** **Descriptive characteristics of the study population** ^**a)**^

	Women (***n*** = 113)		Men (***n*** = 49)		
	Median	P _5_, P _95_	Median	P _5_, P _95_	***P*** ^b)^
25-Hydroxyvitamin D_3_ [nmol/L]^c)^	62.5	39.0 – 87.6	65.6	44.9 – 88.1	0.251
Age (years)	75.0	68.7 – 85.6	76.0	67.5 – 85.0	0.196
Body mass index (kg/m^2^)	27.1	21.1 – 35.0	26.4	21.7 – 33.9	0.792
Total body fat (%)	42.7	32.1 – 50.6	30.9	21.7 – 43.3	< 0.001
Glomerular filtration rate (mL/min/1.73 m^2^)	55.7	39.4 – 69.1	61.5	41.3 – 79.9	< 0.001
Intact parathyroid hormone [pmol/L]	4.6	2.6 – 12.1	4.4	2.1 – 10.6	0.717
Blood sampling in September (*n*, %)	43	38.1	17	34.7	0.726
Time spent outdoors (min/d)	120	37 – 360	150	30 – 540	0.033
Dietary vitamin D intake (μg/d)	3.0	0.4 – 10.3	5.1	0.8 – 19.6	0.099
Vitamin D supplement users (*n*, %)	14	12.4	3	6.1	0.278
Dietary calcium intake (g/d)	1.0	0.5 – 1.7	1.0	0.5 – 1.6	0.969
Calcium supplement users (*n*, %)	41	36.3	9	18.4	0.027
Dietary phosphorus intake (g/d)	1.2	0.8 – 2.1	1.4	1.0 – 2.5	0.006
Alcohol intake (g/d)	0.5	0.0 – 21.7	5.3	0.0 – 28.5	0.010
Physical activity level	1.7	1.5 – 2.0	1.6	1.5 – 1.9	0.066
Current or ex-smokers (*n*, %)	29	25.7	33	67.3	< 0.001
Household income ≥ 1500 €/month (*n*, %)	62	54.9	39	79.6	0.004
Osteoporosis diagnosis/current medication (*n*, %)	48	42.5	4	8.2	< 0.001

^a)^Data are presented as median, 5th and 95th percentiles for continuous variables and absolute and relative frequencies for categorical variables.

^b)^Mann-Whitney *U* test, Chi-square test and Fischer’s exact test for analysing sex differences.

^c)^To convert nmol/L in μg/L, divide by 2.496.

Median vitamin D status and 5th and 95th percentiles according to characteristics of the study population are shown in Table 2. Lower 25(OH)D_3_ concentrations were observed in overweight/obese subjects, subjects with hyperparathyroidism as well as subjects with a household net income < 1500 € per month. In addition, current/ex-smokers and subjects aged 76 and older showed a trend towards lower 25(OH)D_3_ concentrations.

**Table 2:** **Vitamin D status according to selected characteristics of the study population** ^**a)**^

	25-Hydroxyvitamin D _3_ [nmol/L]		
	Median	P _5_, P _95_	***P*** ^ b)^
Age < 76 years (*n* = 82)	65.6	33.8 – 89.9	0.109
Age ≥ 76 years (*n* = 80)	61.2	43.1 – 79.2	
Body mass index < 25 kg/m^2^ (*n* = 51)	67.3	45.0 – 89.4	0.015
Body mass index ≥ 25 kg/m^2^ (*n* = 111)	60.7	38.5 – 84.3	
Blood sampling in July/August (*n* = 102)	61.6	38.0 – 84.1	0.280
Blood sampling in September (*n* = 60)	66.0	44.5 – 90.0	
Vitamin D supplement non users (*n* = 145)	62.9	39.7 – 87.2	0.239
Vitamin D supplement users (*n* = 17)	67.4	32.8 – .	
Intact parathyroid hormone ≤ 6.9 pmol/L (*n* = 138)	64.2	45.3 – 88.5	0.001
Intact parathyroid hormone > 6.9 pmol/L^c)^ (*n* = 24)	51.7	30.3 – 78.2	
eGFR < 60 mL/min/1.73 m^2^ (*n* = 99)	61.4	39.6 – 87.3	0.297
eGFR ≥ 60 mL/min/1.73 m^2^ (*n* = 63)	65.6	39.2 – 88.2	
Never-smokers (*n* = 100)	64.0	39.6 – 88.8	0.162
Current or ex-smokers (*n* = 62)	61.7	39.3 – 86.6	
Household income < 1500 €/month (*n* = 61)	56.9	39.6 – 84.3	0.038
Household income ≥ 1500 €/month (*n* = 101)	65.6	39.4 – 90.0	
Osteoporosis diagnosis/current medication (*n* = 52)	64.3	39.9 – 91.7	0.346
No osteoporosis diagnosis/current medication (*n* = 110)	62.2	38.5 – 85.0	

*Abbreviations*: eGFR, estimated glomerular filtration rate.

^a)^Data are presented as median, 5th and 95th percentiles.

^b)^Mann-Whitney *U* test for analysing group differences.

^c)^Intact parathyroid hormone concentrations > 6.9 pmol/L indicate hyperparathyroidism.

### Impact of disease histories and current medication on serum 25-hydroxyvitamin D_3_ concentrations

Subjects with and without a reported lifetime diagnosis of selected diseases (hypertension, cardiac disease, dyslipidaemia, diabetes mellitus, osteoporosis, cancer, liver/gall bladder disease and urinary tract/kidney disease) and/or current medication for such diseases did not significantly differ in their 25(OH)D_3_ concentrations (data not shown). In linear regression analyses, neither self-reported disease histories nor related medication were significantly associated with 25(OH)D_3_ concentrations before and after adjusting for sex, age, iPTH, % TBF, month of blood sampling, time spent outdoors, PAL, vitamin D intake, use of vitamin D supplements, calcium intake, alcohol consumption and smoking (data not shown), except for intake of osteoporosis drugs, which was associated with higher 25(OH)D_3_ concentrations before (*β* = 0.159; *P* = 0.043), but not after multiple adjustments (*β* = 0.125; *P* = 0.069). Intake of hormones and corticoids showed no associations with 25(OH)D_3_ concentrations before and after adjustments, respectively (data not shown). Therefore, only the variable ‘lifetime history of osteoporosis and/or current osteoporosis medication’ was considered as a potential determinant of the 25(OH)D_3_ concentration in the subsequent multiple regression analyses.

### Independent determinants of 25-hydroxyvitamin D_3_ in the combined study population and stratified by sex

Table 3 shows the results of the multiple regression analyses with the 25(OH)D_3_ concentration as the dependent variable. In the pooled analysis of women and men combined, iPTH, % TBF and smoking were independent negative determinants of the 25(OH)D_3_ concentration, whereas time spent outdoors, blood sampling in September, PAL and intake of vitamin D supplements were significant positive determinants. When iPTH was omitted from the analysis, the results in view of the other predictor variables remained almost unchanged (data not shown). Except for intake of vitamin D supplements, which showed a borderline significant association with 25(OH)D_3_ concentrations (*P* = 0.06), and the adjusted coefficient of determination (*R*^*2*^) of the final regression model was reduced to 0.204. When interaction terms were added separately to the regression model, effect modification by sex was observed with regard to the association of smoking behaviour and income with 25(OH)D_3_, respectively (all *P* < 0.05).

**Table 3:** **Independent predictors of 25(OH)D**_**3**_ **concentrations in the combined population and in women and men separately** ^**a)**^

	Combined sexes (***n*** = 162) ^b)^			Women (***n*** = 113) ^c)^			Men (***n*** = 49) ^c),d)^		
	25(OH)D _3_ [nmol/L]			25(OH)D _3_ [nmol/L]			25(OH)D _3_ [nmol/L]		
	***B***	***β***	***P***	***B***	***β***	***P***	***B***	***β***	***P***
Intercept	50.547		< 0.0001	84.061		< 0.0001	9.646		0.654
Intact parathyroid hormone [pmol/L]	-1.324	-0.286	< 0.0001	-1.558	-0.323	0.0001	-1.041	-0.254	0.043
Total body fat (%)	-0.398	-0.224	0.002	-0.549	-0.208	0.013			
Smoking (no/yes)	-4.291	-0.151	0.030				-7.501	-0.282	0.026
Time spent outdoors (min/d)	0.027	0.242	0.001	0.041	0.328	0.0002			
Month of blood sampling (July/August vs. September)	5.946	0.208	0.003	6.731	0.229	0.007			
Physical activity level	17.489	0.189	0.008				35.623	0.336	0.011
Vitamin D supplements (no/yes)	6.782	0.151	0.028	7.454	0.172	0.040			
Household income ≥ 1500 €/month (no/yes)							9.362	0.302	0.020
Adjusted *R* ^*2*^ for the final model		0.280			0.296			0.316	

*Abbreviations*: 25(OH)D_3_, 25-hydroxyvitamin D_3_; adjusted *R* ^*2*^, proportion of the total variance explained by the final model.

^a)^Multiple linear regression analyses were performed using stepwise backward procedure and the 25(OH)D_3_ concentration as the dependent variable. The results of the analyses are expressed in terms of the non-standardised coefficient beta (*B*), standardised coefficient beta (*β*) and the adjusted coefficient of determination (*R* ^*2*^) for the final model. Only variables, which remained in the final model, are presented.

^b)^Serum 25(OH)D_3_ concentration as dependent variable and age, sex, total body fat, estimated glomerular filtration rate, intact parathyroid hormone, month of blood sampling, time spent outdoors, intake of dietary vitamin D, vitamin D supplements, dietary calcium, calcium supplements, phosphorus and alcohol, physical activity level, smoking and household net income as possible independent variables.

^c)^Serum 25(OH)D_3_ concentration as dependent variable and total body fat, intact parathyroid hormone, month of blood sampling, time spent outdoors, intake of vitamin D supplements, physical activity level, smoking and household net income as possible independent variables.

^d)^When only intact parathyroid hormone, physical activity level, smoking and household net income were included in the male regression model, the results remained unchanged.

In subsequent sex-specific multiple regression analyses (Table 3), those variables which were identified as determinants in the combined population as well as factors for which an interaction by sex was observed were considered as independent variables, i.e. iPTH, % TBF, time spent outdoors, month of blood sampling, smoking behaviour, PAL, intake of vitamin D supplements and household income. In women, iPTH and % TBF were independent negative determinants of the 25(OH)D_3_ concentration, whereas time spent outdoors, blood sampling in September and intake of vitamin D supplements were identified as positive determinants. In men, iPTH and current or past smoking were negative predictors of the 25(OH)D_3_ concentration, whereas PAL and a monthly household net income ≥ 1500 € were positive determinants. The respective regression models accounted for 28%, 30% and 32% of the variance in 25(OH)D_3_ concentrations in the combined study population, in women and in men, respectively. However, iPTH was no significant predictor in men after excluding one male subject with an iPTH value of 21 pmol/L.

The results of the multiple regression analyses (Table 3) remained unchanged when lifetime diagnosis of osteoporosis/current osteoporosis medication was considered as a covariate. When analyses were repeated by using the square root transformed 25(OH)D_3_ concentrations, the results regarding the independent predictors of 25(OH)D_3_ were almost identical (data not shown), but the regression models explained slightly higher proportions of the variance in 25(OH)D_3_ in the combined study population (*R*^*2*^ = 0.285) and in women (*R*^*2*^ = 0.304), but not in men (*R*^*2*^ = 0.313).

Finally, we performed a sensitivity analysis excluding subjects with an eGFR ≤ 30 mL/min/1.73 m^2^ (*n* = 3). By doing this, the results with regard to the determinants of the 25(OH)D_3_ concentration remained essentially unchanged (data not shown).

## Discussion

The findings of the present study indicate that serum 25(OH)D_3_ concentrations are influenced by body composition, month of blood sampling, socioeconomic factors, lifestyle variables, and iPTH, but may not be associated with sex, age, eGFR, dietary intake of vitamin D, calcium, phosphorus and alcohol, and selected disease histories or medication in community-dwelling elderly subjects. Although vitamin D status did not significantly differ between sexes, our results provide evidence for sex-specific determinants of the 25(OH)D_3_ concentration. In women, iPTH, body composition, sun exposure (reflected by month of blood sampling and time spent outdoors) and intake of vitamin D supplements were the predominant predictors, whereas in men, iPTH, smoking, PAL and household net income predicted 25(OH)D_3_ concentrations. However, the impact of iPTH on 25(OH)D_3_ in men relied on the inclusion of one subject with a particularly high iPTH value. Findings from previous studies also point to sex differences in predictors of 25(OH)D concentrations [7, 11, 18], but in these studies, categorised variables were primarily investigated and interaction terms were not specifically addressed. In addition, BMI and not % TBF was considered as potential determinant, which might be critical considering that women exhibited a higher % TBF for a given BMI than men [29] and that the % TBF has been proven as a stronger determinant of 25(OH)D_3_ than anthropometric variables [13].

In contrast to some previous studies in elderly Europeans [30–32], no participant of the GISELA study had a severe vitamin D deficiency and only 19% showed 25(OH)D_3_ concentrations below 50 nmol/L. This may be partially due to the fact that our study is restricted to the summer months and that our subjects were above-average educated and health-conscious volunteers. There is evidence that the assessment of the 25(OH)D concentration by immunoassays may result in an overestimation of the prevalence of vitamin D deficiency when standardisation against LC-MS/MS is missing [31]; however, the 25(OH)D_3_ assay used in the present study has been standardised against LC-MS/MS. The prevalence of vitamin D insufficiency increased to 81% when applying the higher cut-off value of 75 nmol/L. Therefore, the classification and, consequently, the interpretation may depend on both the respective assay and the cut-off value used.

Serum iPTH was one of the strongest negative predictors of 25(OH)D_3_ in our study population. The inverse relationship between iPTH and 25(OH)D has been frequently reported in the literature [30, 33], and may function in both directions: Low 25(OH)D_3_ concentrations may result in low serum calcium concentrations followed by a stimulation of iPTH secretion, which in turn promotes the production of 1,25-dihydroxyvitamin D_3_ [1,25(OH)_2_D_3_] and consequently increases the turnover of 25(OH)D_3_ [34, 35].

In agreement with previous findings [9, 11–14, 36], our results demonstrate that compared with lean subjects, overweight and obese individuals have significantly lower 25(OH)D_3_ concentrations. However, there is still a debate on the underlying mechanism as regards this association. Several theories have been proposed. The most popular are: 1) sequestration of vitamin D by fat cells [37]; 2) volumetric dilution effect [36]; 3) sedentary lifestyle [38]; and finally, 4) reverse causality [39]. In the present study, the inverse association between % TBF and 25(OH)D_3_ was independent of lifestyle factors and predominantly found in women, possibly due to the higher amount of TBF in women than in men [13].

With regard to lifestyle factors, we found associations of time spent outdoors, PAL and smoking with 25(OH)D_3_ regardless of body composition. In this context, sex may act as an effect modifier. The time spent outdoors seems to have an effect on 25(OH)D_3_ predominantly in women, whereas physical activity and smoking predicted 25(OH)D_3_ in men. According to the results of previous studies, especially outdoor activities, such as cycling and gardening, are associated with a lower likelihood of an inadequate vitamin D status in elderly people [32, 40]. We did not distinguish physical activities by type or location, so the observed sex differences in our study may be attributed to sex-specific physical activity pattern and differences in the intensity, time of day and clothing while engaged in activities. For example, female subjects of the GISELA study spent more time on household chore than men (Median: 120 vs. 34 min/d; *P* < 0.001), whereas men spent significantly more daily time on gardening than women (Median: 43 vs. 17 min/d; *P* < 0.05). One may speculate that PAL reflects the UVB exposure to some extent analogous to the reported time spent outdoors and effects of both parameters in one regression model may cancel each other out. However, collinearity was not found in our study. This indicates that independent effects might exist as in the total population both parameters proved as independent predictors. These findings are in line with a cross-sectional study of 644 subjects aged 60 to 84 years, in which the association between physical activity and 25(OH)D was independent of time spent outdoors and also only found in men [18]. Furthermore, a prospective study over a mean follow-up of 2.6 years in 686 community-dwelling older adults demonstrated that higher physical activity increased 25(OH)D concentration after adjusting for sun exposure [41]. While the positive impact of sun exposure on vitamin D levels is well documented, the role of physical activity and smoking is less well understood. Some studies reported significant associations [14, 42, 43], but especially the association between smoking and 25(OH)D was frequently not found in other studies [7, 10, 18]. The smaller proportion of female smokers in the present study may have caused the missing impact of smoking on 25(OH)D_3_ concentrations in women. Apart from the suggested indirect effects of physical activity on vitamin D through sun exposure and changes in body composition [7, 40], physical activity may directly influence vitamin D by changes in calciotropic hormones or by increasing concentrations of insulin-like growth factor-I [32, 44, 45]. Whether smoking has a direct effect on the vitamin D metabolism is unclear. There is some evidence that smoking may affect the synthesis of 25(OH)D by inhibiting the expression of cytochrome P_450_ 2R1 in the liver [46]. In addition, benzo[a]pyrene, which is produced by cigarette combustion, may exaggerate the degradation of 25(OH)D_3_ and 1,25(OH)_2_D_3_ by stimulating the 1,25(OH)_2_D_3_-dependent induction of cytochrome P_450_ 24A1 [47].

Economic aspects affected the vitamin D status in our subjects, inasmuch as we found an association between household income and 25(OH)D_3_ concentrations in men. Women showed no significant association, possibly because they generally reported lower incomes. Some previous studies also indicated a positive influence of a high income on 25(OH)D_3_ [48], whereas others could not confirm an association [38, 43]. As the classification of income and the population characteristics vary widely between studies, direct comparisons of study results are difficult. The majority of previous studies as well as our study did not analyse individual but household income, what may bias the association. It can be assumed that the household income might partially reflect the marital status of subjects.

Dietary factors, such as intake of vitamin D, calcium, phosphorus and alcohol did not affect 25(OH)D_3_ concentrations in our study cohort. In contrast, several previous studies have confirmed vitamin D intake as an independent predictor of 25(OH)D concentrations [11, 12, 15]. The missing association between vitamin D intake and 25(OH)D_3_ concentrations in our study may be due to the facts that we distinguished between dietary and supplemental vitamin D and food fortification is not as much common in Germany as in the US and Canada. Other European studies also found no significant association between dietary vitamin D and 25(OH)D [14, 31]. Furthermore, our study was restricted to the summer, when the effect of the low habitual vitamin D intake may be masked by the contribution of daily sun exposure to 25(OH)D_3_ concentrations. However, the use of vitamin D supplements resulted in higher 25(OH)D_3_ concentrations of about 7 nmol/L, despite the small proportion of subjects who took vitamin D supplements. The effect was predominant in women, most likely because only three male subjects reported an intake of vitamin D supplements. With the exception of one male subject, all vitamin D supplement users also consumed calcium supplements. This makes it difficult to distinguish between the individual effects of the two supplements, and could indicate a treatment of osteoporosis [49]. In this context, we want to emphasise that lifetime diagnosis of osteoporosis as well as the other investigated chronic diseases along with current medication showed no influence on 25(OH)D_3_ concentrations in our cohort. Some researchers [42] suggested that the observed associations between chronic diseases and 25(OH)D concentrations are explained by non-disease factors, such as PAL and sun exposure, and thus do not represent causal associations. The missing associations in our study population may be because subjects with and without reported lifetime history of selected diseases and/or accompanying medication did not considerably differ in lifestyle factors, such as time spent outdoors, PAL and vitamin D intake (data not shown). Furthermore, some diseases and medication were rarely reported by our participants, such as diabetes and insulin or cancer and cytostatic drugs (data not shown).

In summary, we considered a variety of potential determinants of 25(OH)D_3_, but the final regression model only accounted for 28.5% of the variance in 25(OH)D_3_ concentrations in the combined study population. Slightly higher percentages were observed when sex-specific analyses were performed, resulting in a *R*^*2*^ of 0.30 in women and 0.32 in men. Previous studies [7, 11, 12, 15, 18, 48] often explained comparable or somewhat lower proportions of between-subject variance in 25(OH)D concentrations. The relatively low explanatory power may be attributed to the indirect assessment of lifestyle factors, but may also result from other factors, such as genetics, serum calcium concentration and skin type. However, even in studies with an evaluation of genetic factors and a more detailed assessment on individual sun exposure, only 54% of the variation in 25(OH)D concentrations were explained, whereupon 36% and 14% were due to sun exposure related factors and genetic factors, respectively [50]. Consequently, up to now, the identified predictors of 25(OH)D cannot be considered as reliable surrogate parameters of the individual 25(OH)D concentration [15], but may help to identify subjects at risk for vitamin D insufficiency.

A main strength of our study is that measurements of 25(OH)D_3_ were performed in a single batch and standardised against LC-MS/MS. Additional strengths are the recruitment of subjects from a single geographic area and the consideration of a variety of potential determinants, including also health aspects, and the fact that our study population, which exclusively comprised subjects > 65 years, was free of a severe vitamin D deficiency. Seasonal and diurnal variations in serum 25(OH)D_3_ concentrations were minimised by blood sampling in the morning hours from July to September. Main limitations of the present study lie in the cross-sectional nature, the relatively small sample size and the fact that the GISELA cohort is not a nationally representative sample. In view of the sample size, the statistical power might be too low to detect moderate associations, particularly in men. Individual serum 25(OH)D_3_ concentrations were only measured once and thus, might not reflect year-long vitamin D status. Because information on skin type, clothing habits, sun protection practices, past vacations in sunny areas and time of day when subjects were outside was not assessed, precise estimation of individual UVB exposition is difficult. In addition, we relied on self-reported data concerning dietary intake, lifestyle factors, income and health status. Finally, information on reason, type, dosage and duration of vitamin D supplementation was not available.

## Conclusion

Elderly people living in private households can generally achieve a vitamin D status > 50 nmol/L in the summer months when they follow an active and health conscious lifestyle, including outdoor activities of approximately two hours a day, abstain from smoking and maintain a normal body weight. Thus, vitamin D status is modifiable throughout behavioural interventions. Although vitamin D status did not significantly differ between sexes, sex-specific determinants of 25(OH)D_3_ concentrations seem to exist. While iPTH was a negative predictor of vitamin D in both sexes, % TBF, sun exposure and intake of supplements had a greater impact in women, whereas smoking, PAL and monthly household net income predicted 25(OH)D_3_ concentrations predominantly in men. This should be considered when recommendations to improve vitamin D status are implemented.

## Abbreviations

25(OH)D: 25-Hydroxyvitamin D
25(OH)D_3_: 25-Hydroxyvitamin D_3_
1: 25(OH)_2_D_3_: 1,25-Dihydroxyvitamin D_3_
*B*: Non-standardised coefficient beta
*β*: Standardised coefficient beta
BMI: Body mass index
ECLIA: Electrochemiluminescence immunoassay
eGFR: Estimated glomerular filtration rate
GISELA: Longitudinal study on nutrition and health status of senior citizens in Giessen, Germany
iPTH: Intact parathyroid hormone
LC-MS/MS: Liquid chromatography-tandem mass spectrometry
PAL: Physical activity level
*R*^*2*^: Adjusted coefficient of determination
RMR: Resting metabolic rate
TBF: Total body fat
WHO: World Health Organization.

## References

[1] Bruyère O, Slomian J, Beaudart C, Buckinx F, Cavalier E, Gillain S (2014). Prevalence of vitamin D inadequacy in European women aged over 80 years. Arch Gerontol Geriatr.

[2] Feldman D, Krishnan AV, Swami S, Giovannucci E, Feldman BJ (2014). The role of vitamin D in reducing cancer risk and progression. Nat Rev Cancer.

[3] Norman PE, Powell JT (2014). Vitamin D and cardiovascular disease. Circ Res.

[4] Hirani V, Cumming RG, Le Couteur DG, Naganathan V, Blyth F, Handelsman DJ (2014). Low levels of 25-hydroxyvitamin D and active 1,25-dihydroxyvitamin D independently associated with type 2 diabetes mellitus in older Australian men: the Concord Health and Ageing in Men Project. J Am Geriatr Soc.

[5] Ross AC, Manson JE, Abrams SA, Aloia JF, Brannon PM, Clinton SK (2011). The 2011 report on dietary reference intakes for calcium and vitamin D from the Institute of Medicine: what clinicians need to know. J Clin Endocrinol Metab.

[6] Holick MF, Binkley NC, Bischoff-Ferrari HA, Gordon CM, Hanley DA, Heaney RP (2011). Evaluation, treatment, and prevention of vitamin D deficiency: an Endocrine Society clinical practice guideline. J Clin Endocrinol Metab.

[7] Freedman DM, Cahoon EK, Rajaraman P, Major JM, Doody MM, Alexander BH (2013). Sunlight and other determinants of circulating 25-hydroxyvitamin D levels in black and white participants in a nationwide U.S. study. Am J Epidemiol.

[8] McCarty CA (2008). Sunlight exposure assessment: can we accurately assess vitamin D exposure from sunlight questionnaires?. Am J Clin Nutr.

[9] Hirani V, Tull K, Ali A, Mindell J (2010). Urgent action needed to improve vitamin D status among older people in England!. Age Ageing.

[10] Daly RM, Gagnon C, Lu ZX, Magliano DJ, Dunstan DW, Sikaris KA (2012). Prevalence of vitamin D deficiency and its determinants in Australian adults aged 25 years and older: a national, population-based study. Clin Endocrinol (Oxf).

[11] McCullough ML, Weinstein SJ, Freedman DM, Helzlsouer K, Flanders WD, Koenig K (2010). Correlates of circulating 25-hydroxyvitamin D: Cohort Consortium Vitamin D Pooling Project of Rarer Cancers. Am J Epidemiol.

[12] Bertrand KA, Giovannucci E, Liu Y, Malspeis S, Eliassen AH, Wu K (2012). Determinants of plasma 25-hydroxyvitamin D and development of prediction models in three US cohorts. Br J Nutr.

[13] Jungert A, Roth HJ, Neuhäuser-Berthold M (2012). Serum 25-hydroxyvitamin D_3_ and body composition in an elderly cohort from Germany: a cross sectional study. Nutr Metab (Lond).

[14] Thuesen B, Husemoen L, Fenger M, Jakobsen J, Schwarz P, Toft U (2012). Determinants of vitamin D status in a general population of Danish adults. Bone.

[15] Millen AE, Wactawski-Wende J, Pettinger M, Melamed ML, Tylavsky FA, Liu S (2010). Predictors of serum 25-hydroxyvitamin D concentrations among postmenopausal women: the Women’s Health Initiative Calcium plus Vitamin D clinical trial. Am J Clin Nutr.

[16] Gröber U, Kisters K (2012). Influence of drugs on vitamin D and calcium metabolism. Dermatoendocrinol.

[17] Sohl E, van Schoor NM, de Jongh RT, de Vries OJ, Lips P (2012). The impact of medication on vitamin D status in older individuals. Eur J Endocrinol.

[18] Tran B, Armstrong BK, McGeechan K, Ebeling PR, English DR, Kimlin MG (2013). Predicting vitamin D deficiency in older Australian adults. Clin Endocrinol (Oxf).

[19] Roche Diagnostics GmbH (2007). Elecsys and cobas analyzer. Vitamin D3 (25-OH).

[20] Roche Diagnostics GmbH (2003). Elecsys 1010/2010/Modular analytics E170. PTH.

[21] Seelig HP, Wüst H (1969). Die Kreatininbestimmung mit der Jaffé Reaktion. Ärztl Lab.

[22] Levey AS, Greene T, Kusek J, Beck G (2000). A simplified equation to predict glomerular filtration rate from serum creatinine (abstract). J Am Soc Nephrol.

[23] Roubenoff R, Baumgartner RN, Harris TB, Dallal GE, Hannan MT, Economos CD (1997). Application of bioelectrical impedance analysis to elderly populations. J Gerontol A Biol Sci Med Sci.

[24] WHO (World Health Organization) (1985). Energy and protein requirements.

[25] Weir JB (1949). New methods for calculating metabolic rate with special reference to protein metabolism. J Physiol.

[26] Krems C, Lührmann PM, Neuhäuser-Berthold M (2004). Physical activity in young and elderly subjects. J Sports Med Phys Fitness.

[27] Federal Office for Radiation Protection: **Solare bodennahe UV-Strahlung in Deutschland (Solar ground-level UV radiation in Germany).** [ Accessed August 26th 2014 http://www.bfs.de/de/uv/uv2/uv_messnetz/Abb12_2008.pdf]

[28] Lührmann PM, Herbert B, Gaster C, Neuhäuser-Berthold M (1999). Validation of a self-administered 3-day estimated dietary record for use in the elderly. Eur J Nutr.

[29] Pasco JA, Nicholson GC, Brennan SL, Kotowicz MA (2012). Prevalence of obesity and the relationship between the body mass index and body fat: cross-sectional, population-based data. PLoS One.

[30] Houston DK, Cesari M, Ferrucci L, Cherubini A, Maggio D, Bartali B (2007). Association between vitamin D status and physical performance: the InCHIANTI study. J Gerontol A Biol Sci Med Sci.

[31] Perna L, Haug U, Schöttker B, Müller H, Raum E, Jansen EH (2012). Public health implications of standardized 25-hydroxyvitamin D levels: a decrease in the prevalence of vitamin D deficiency among older women in Germany. Prev Med.

[32] van den Heuvel EG, van Schoor N, de Jongh RT, Visser M, Lips P (2013). Cross-sectional study on different characteristics of physical activity as determinants of vitamin D status; inadequate in half of the population. Eur J Clin Nutr.

[33] Sai AJ, Walters RW, Fang X, Gallagher JC (2011). Relationship between vitamin D, parathyroid hormone, and bone health. J Clin Endocrinol Metab.

[34] Lips P (2001). Vitamin D deficiency and secondary hyperparathyroidism in the elderly: consequences for bone loss and fractures and therapeutic implications. Endocr Rev.

[35] Jones KS, Assar S, Vanderschueren D, Bouillon R, Prentice A, Schoenmakers I: **Predictors of 25(OH)D half-life and plasma 25(OH)D concentration in the Gambia and the UK.** *Osteoporos Int* in press 10.1007/s00198-014-2905-0 PMC4331602 25278297

[36] Drincic AT, Armas LA, Van Diest EE, Heaney RP (2012). Volumetric dilution, rather than sequestration best explains the low vitamin D status of obesity. Obesity (Silver Spring).

[37] Wortsman J, Matsuoka LY, Chen TC, Lu Z, Holick MF (2000). Decreased bioavailability of vitamin D in obesity. Am J Clin Nutr.

[38] Hirani V, Cumming RG, Blyth FM, Naganathan V, Le Couteur DG, Handelsman DJ (2013). Vitamin D status among older community dwelling men living in a sunny country and associations with lifestyle factors: the Concord Health and Ageing in Men Project, Sydney, Australia. J Nutr Health Aging.

[39] Ding C, Gao D, Wilding J, Trayhurn P, Bing C (2012). Vitamin D signalling in adipose tissue. Br J Nutr.

[40] De Rui M, Toffanello ED, Veronese N, Zambon S, Bolzetta F, Sartori L (2014). Vitamin D deficiency and leisure time activities in the elderly: are all pastimes the same?. PLoS One.

[41] Scott D, Blizzard L, Fell J, Ding C, Winzenberg T, Jones G (2010). A prospective study of the associations between 25-hydroxyvitamin D, sarcopenia progression and physical activity in older adults. Clin Endocrinol (Oxf).

[42] Cheng TY, Millen AE, Wactawski-Wende J, Beresford SA, LaCroix AZ, Zheng Y (2014). Vitamin D intake determines vitamin D status of postmenopausal women, particularly those with limited sun exposure. J Nutr.

[43] Miettinen ME, Kinnunen L, Leiviskä J, Keinänen-Kiukaanniemi S, Korpi-Hyövälti E, Niskanen L (2014). Association of serum 25-hydroxyvitamin D with lifestyle factors and metabolic and cardiovascular disease markers: population-based cross-sectional study (FIN-D2D). PLoS One.

[44] Gómez JM, Maravall FJ, Gómez N, Navarro MA, Casamitjana R, Soler J (2004). Relationship between 25(OH)D_3_, the IGF-I system, leptin, anthropometric and body composition variables in a healthy, randomly selected population. Horm Metab Res.

[45] Maïmoun L, Sultan C (2009). Effect of physical activity on calcium homeostasis and calciotropic hormones: a review. Calcif Tissue Int.

[46] O’Shaughnessy PJ, Monteiro A, Bhattacharya S, Fowler PA (2011). Maternal smoking and fetal sex significantly affect metabolic enzyme expression in the human fetal liver. J Clin Endocrinol Metab.

[47] Matsunawa M, Amano Y, Endo K, Uno S, Sakaki T, Yamada S (2009). The aryl hydrocarbon receptor activator benzo[a]pyrene enhances vitamin D_3_ catabolism in macrophages. Toxicol Sci.

[48] Martini LA, Verly E, Marchioni DM, Fisberg RM (2013). Prevalence and correlates of calcium and vitamin D status adequacy in adolescents, adults, and elderly from the Health Survey-São Paulo. Nutrition.

[49] Cashman KD, Muldowney S, McNulty B, Nugent A, FitzGerald AP, Kiely M (2013). Vitamin D status of Irish adults: findings from the National Adult Nutrition Survey. Br J Nutr.

[50] Lucas RM, Ponsonby AL, Dear K, Valery PC, Taylor B, van der Mei I (2013). Vitamin D status: multifactorial contribution of environment, genes and other factors in healthy Australian adults across a latitude gradient. J Steroid Biochem Mol Biol.

